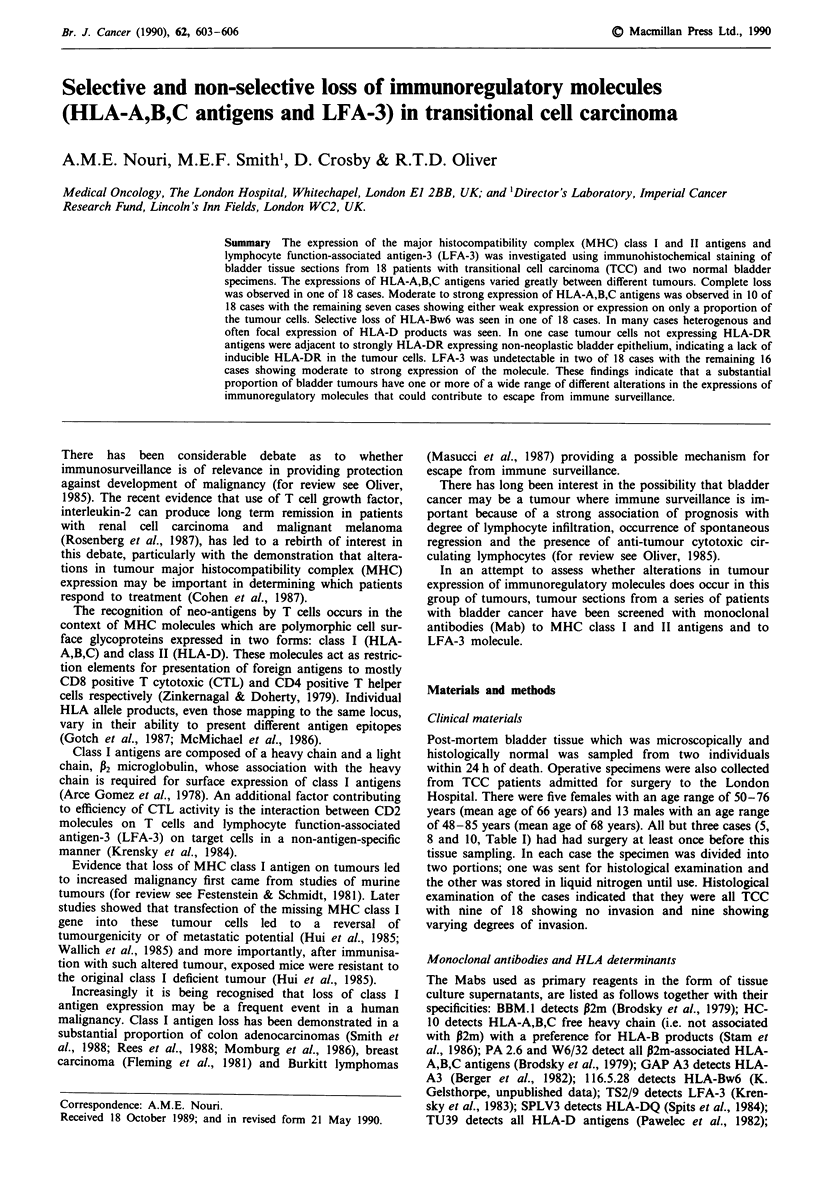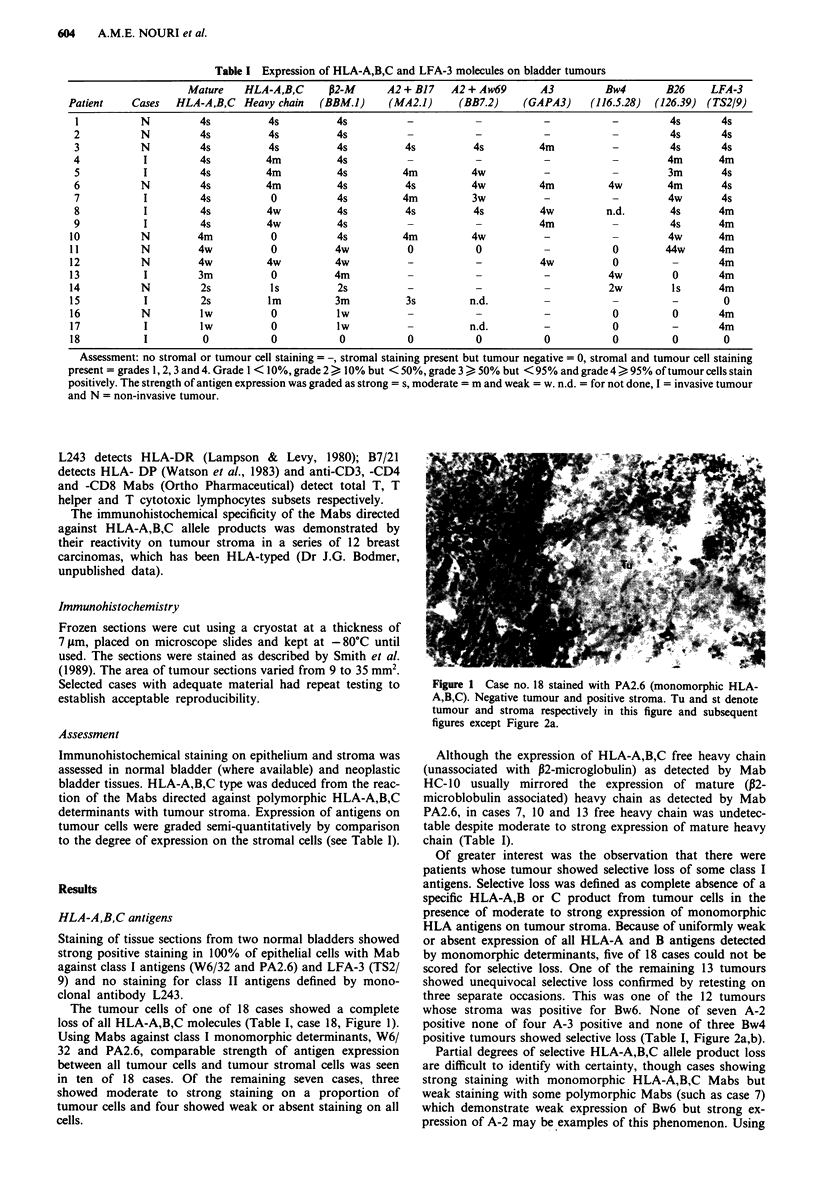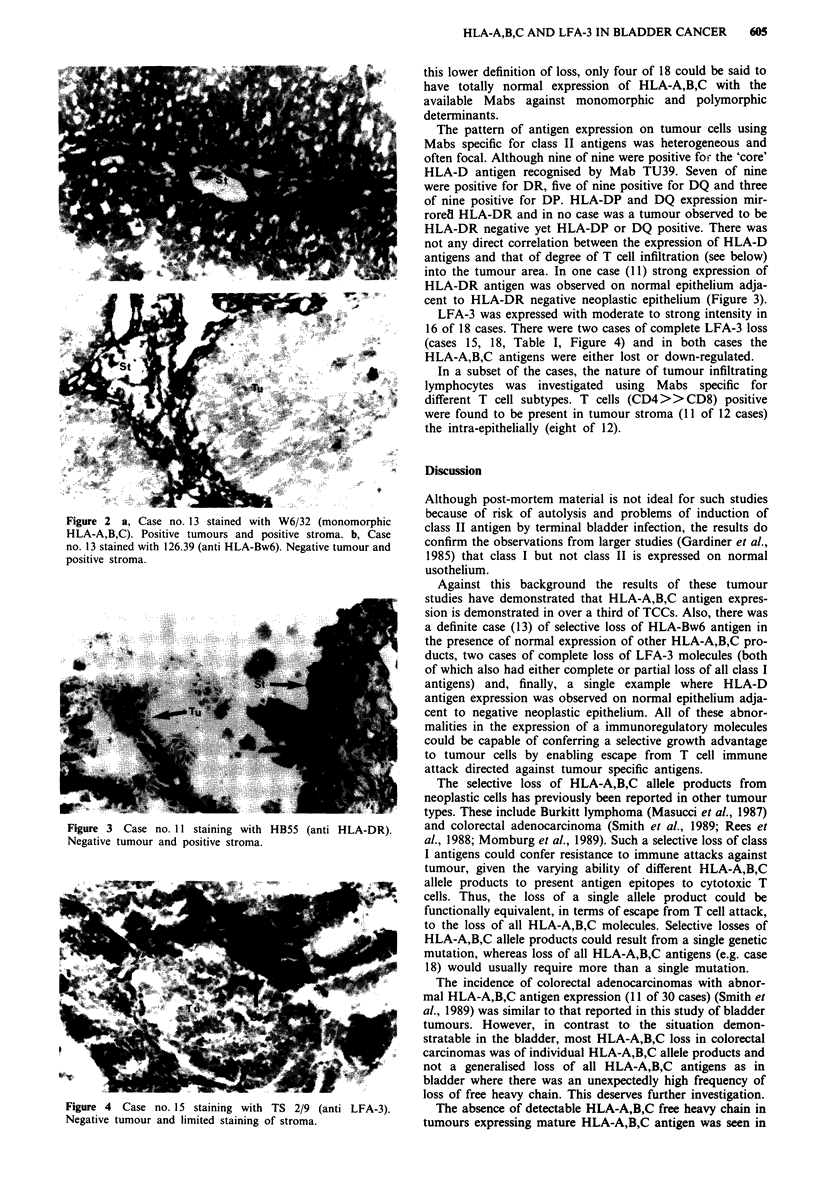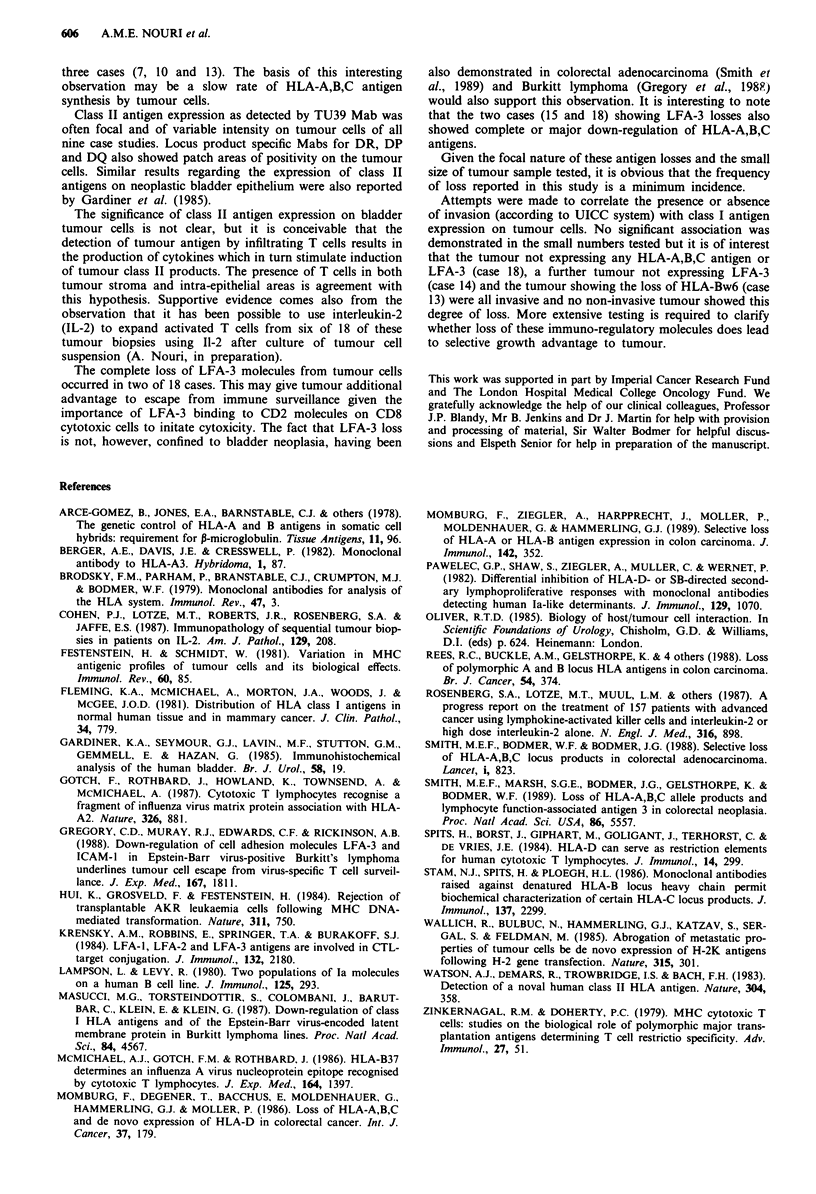# Selective and non-selective loss of immunoregulatory molecules (HLA-A,B,C antigens and LFA-3) in transitional cell carcinoma.

**DOI:** 10.1038/bjc.1990.338

**Published:** 1990-10

**Authors:** A. M. Nouri, M. E. Smith, D. Crosby, R. T. Oliver

**Affiliations:** Medical Oncology, London Hospital, Whitechapel, UK.

## Abstract

**Images:**


Br. J. Cancer (1990), 62, 603-606 © Macmillan Press Ltd., 1990

### Selective and non-selective loss of immunoregulatory molecules (HLA-A,B,C antigens and LFA-3) in transitional cell carcinoma

A.M.E. Nouri, M.E.F. Smith[1], D. Crosby & R.T.D. Oliver

*Medical Oncology, The London Hospital, Whitechapel, London E1 2BB, UK; and [1]Director's Laboratory, Imperial Cancer Research Fund, Lincoln's Inn Fields, London WC2, UK.*

**Summary** The expression of the major histocompatibility complex (MHC) class I and II antigens and lymphocyte function-associated antigen-3 (LFA-3) was investigated using immunohistochemical staining of bladder tissue sections from 18 patients with transitional cell carcinoma (TCC) and two normal bladder specimens. The expressions of HLA-A,B,C antigens varied greatly between different tumours. Complete loss was observed in one of 18 cases. Moderate to strong expression of HLA-A,B,C antigens was observed in 10 of 18 cases with the remaining seven cases showing either weak expression or expression on only a proportion of the tumour cells. Selective loss of HLA-Bw6 was seen in one of 18 cases. In many cases heterogenous and often focal expression of HLA-D products was seen. In one case tumour cells not expressing HLA-DR antigens were adjacent to strongly HLA-DR expressing non-neoplastic bladder epithelium, indicating a lack of inducible HLA-DR in the tumour cells. LFA-3 was undetectable in two of 18 cases with the remaining 16 cases showing moderate to strong expression of the molecule. These findings indicate that a substantial proportion of bladder tumours have one or more of a wide range of different alterations in the expressions of immunoregulatory molecules that could contribute to escape from immune surveillance.

There has been considerable debate as to whether immunosurveillance is of relevance in providing protection against development of malignancy (for review see Oliver, 1985). The recent evidence that use of T cell growth factor, interleukin-2 can produce long term remission in patients with renal cell carcinoma and malignant melanoma (Rosenberg *et al.*, 1987), has led to a rebirth of interest in this debate, particularly with the demonstration that alterations in tumour major histocompatibility complex (MHC) expression may be important in determining which patients respond to treatment (Cohen *et al.*, 1987).

The recognition of neo-antigens by T cells occurs in the context of MHC molecules which are polymorphic cell surface glycoproteins expressed in two forms: class I (HLA-A,B,C) and class II (HLA-D). These molecules act as restriction elements for presentation of foreign antigens to mostly CD8 positive T cytotoxic (CTL) and CD4 positive T helper cells respectively (Zinkernagal & Doherty, 1979). Individual HLA allele products, even those mapping to the same locus, vary in their ability to present different antigen epitopes (Gotch *et al.*, 1987; McMichael *et al.*, 1986).

Class I antigens are composed of a heavy chain and a light chain, $\beta_2$ microglobulin, whose association with the heavy chain is required for surface expression of class I antigens (Arce Gomez *et al.*, 1978). An additional factor contributing to efficiency of CTL activity is the interaction between CD2 molecules on T cells and lymphocyte function-associated antigen-3 (LFA-3) on target cells in a non-antigen-specific manner (Krensky *et al.*, 1984).

Evidence that loss of MHC class I antigen on tumours led to increased malignancy first came from studies of murine tumours (for review see Festenstein & Schmidt, 1981). Later studies showed that transfection of the missing MHC class I gene into these tumour cells led to a reversal of tumourgenicity or of metastatic potential (Hui *et al.*, 1985; Wallich *et al.*, 1985) and more importantly, after immunisation with such altered tumour, exposed mice were resistant to the original class I deficient tumour (Hui *et al.*, 1985).

Increasingly it is being recognised that loss of class I antigen expression may be a frequent event in a human malignancy. Class I antigen loss has been demonstrated in a substantial proportion of colon adenocarcinomas (Smith *et al.*, 1988; Rees *et al.*, 1988; Momburg *et al.*, 1986), breast carcinoma (Fleming *et al.*, 1981) and Burkitt lymphomas (Masucci *et al.*, 1987) providing a possible mechanism for escape from immune surveillance.

There has long been interest in the possibility that bladder cancer may be a tumour where immune surveillance is important because of a strong association of prognosis with degree of lymphocyte infiltration, occurrence of spontaneous regression and the presence of anti-tumour cytotoxic circulating lymphocytes (for review see Oliver, 1985).

In an attempt to assess whether alterations in tumour expression of immunoregulatory molecules does occur in this group of tumours, tumour sections from a series of patients with bladder cancer have been screened with monoclonal antibodies (Mab) to MHC class I and II antigens and to LFA-3 molecule.

### Materials and methods

### *Clinical materials*

Post-mortem bladder tissue which was microscopically and histologically normal was sampled from two individuals within 24 h of death. Operative specimens were also collected from TCC patients admitted for surgery to the London Hospital. There were five females with an age range of 50-76 years (mean age of 66 years) and 13 males with an age range of 48-85 years (mean age of 68 years). All but three cases (5, 8 and 10, Table I) had had surgery at least once before this tissue sampling. In each case the specimen was divided into two portions; one was sent for histological examination and the other was stored in liquid nitrogen until use. Histological examination of the cases indicated that they were all TCC with nine of 18 showing no invasion and nine showing varying degrees of invasion.

### *Monoclonal antibodies and HLA determinants*

The Mabs used as primary reagents in the form of tissue culture supernatants, are listed as follows together with their specificities: BBM.1 detects β2m (Brodsky *et al.*, 1979); HC-10 detects HLA-A,B,C free heavy chain (i.e. not associated with β2m) with a preference for HLA-B products (Stam *et al.*, 1986); PA 2.6 and W6/32 detect all β2m-associated HLA-A,B,C antigens (Brodsky *et al.*, 1979); GAP A3 detects HLA-A3 (Berger *et al.*, 1982); 116.5.28 detects HLA-Bw6 (K. Gelsthorpe, unpublished data); TS2/9 detects LFA-3 (Krensky *et al.*, 1983); SPLV3 detects HLA-DQ (Spits *et al.*, 1984); TU39 detects all HLA-D antigens (Pawelec *et al.*, 1982);

Correspondence: A.M.E. Nouri.
Received 18 October 1989; and in revised form 21 May 1990.

**Table I** Expression of HLA-A,B,C and LFA-3 molecules on bladder tumours

| *Patient* | *Cases* | *Mature HLA-A,B,C* | *HLA-A,B,C Heavy chain* | *β2-M (BBM.1)* | *A2 + B17 (MA2.1)* | *A2 + Aw69 (BB7.2)* | *A3 (GAPA3)* | *Bw4 (116.5.28)* | *B26 (126.39)* | *LFA-3 (TS2/9)* |
|---|---|---|---|---|---|---|---|---|---|---|
| 1 | N | 4s | 4s | 4s | – | – | – | – | 4s | 4s |
| 2 | N | 4s | 4s | 4s | – | – | – | – | 4s | 4s |
| 3 | N | 4s | 4s | 4s | 4s | 4s | 4m | – | 4s | 4s |
| 4 | I | 4s | 4m | 4s | – | – | – | – | 4m | 4m |
| 5 | I | 4s | 4m | 4s | 4m | 4w | – | – | 3m | 4s |
| 6 | N | 4s | 4m | 4s | 4s | 4w | 4m | 4w | 4m | 4s |
| 7 | I | 4s | 0 | 4s | 4m | 3w | – | – | 4w | 4s |
| 8 | I | 4s | 4w | 4s | 4s | 4s | 4w | n.d. | 4s | 4m |
| 9 | I | 4s | 4w | 4s | – | – | 4m | – | 4s | 4m |
| 10 | N | 4m | 0 | 4s | 4m | 4w | – | – | 4w | 4m |
| 11 | N | 4w | 0 | 4w | 0 | 0 | – | 0 | 44w | 4m |
| 12 | N | 4w | 4w | 4w | – | – | 4w | 0 | – | 4m |
| 13 | I | 3m | 0 | 4m | – | – | – | 4w | 0 | 4m |
| 14 | N | 2s | 1s | 2s | – | – | – | 2w | 1s | 4m |
| 15 | I | 2s | 1m | 3m | 3s | n.d. | – | – | – | 0 |
| 16 | N | 1w | 0 | 1w | – | – | – | 0 | 0 | 4m |
| 17 | I | 1w | 0 | 1w | – | n.d. | – | 0 | – | 4m |
| 18 | I | 0 | 0 | 0 | 0 | 0 | 0 | 0 | 0 | 0 |

Assessment: no stromal or tumour cell staining = –, stromal staining present but tumour negative = 0, stromal and tumour cell staining present = grades 1, 2, 3 and 4. Grade 1 < 10%, grade 2 ⩾ 10% but < 50%, grade 3 ⩾ 50% but < 95% and grade 4 ⩾ 95% of tumour cells stain positively. The strength of antigen expression was graded as strong = s, moderate = m and weak = w. n.d. = for not done, I = invasive tumour and N = non-invasive tumour.

L243 detects HLA-DR (Lampson & Levy, 1980); B7/21 detects HLA- DP (Watson *et al.*, 1983) and anti-CD3, -CD4 and -CD8 Mabs (Ortho Pharmaceutical) detect total T, T helper and T cytotoxic lymphocytes subsets respectively.

The immunohistochemical specificity of the Mabs directed against HLA-A,B,C allele products was demonstrated by their reactivity on tumour stroma in a series of 12 breast carcinomas, which has been HLA-typed (Dr J.G. Bodmer, unpublished data).

*Immunohistochemistry*

Frozen sections were cut using a cryostat at a thickness of 7 μm, placed on microscope slides and kept at −80°C until used. The sections were stained as described by Smith *et al.* (1989). The area of tumour sections varied from 9 to 35 $mm^2$. Selected cases with adequate material had repeat testing to establish acceptable reproducibility.

*Assessment*

Immunohistochemical staining on epithelium and stroma was assessed in normal bladder (where available) and neoplastic bladder tissues. HLA-A,B,C type was deduced from the reaction of the Mabs directed against polymorphic HLA-A,B,C determinants with tumour stroma. Expression of antigens on tumour cells were graded semi-quantitatively by comparison to the degree of expression on the stromal cells (see Table I).

### Results

*HLA-A,B,C antigens*

Staining of tissue sections from two normal bladders showed strong positive staining in 100% of epithelial cells with Mab against class I antigens (W6/32 and PA2.6) and LFA-3 (TS2/9) and no staining for class II antigens defined by monoclonal antibody L243.

The tumour cells of one of 18 cases showed a complete loss of all HLA-A,B,C molecules (Table I, case 18, Figure 1). Using Mabs against class I monomorphic determinants, W6/32 and PA2.6, comparable strength of antigen expression between all tumour cells and tumour stromal cells was seen in ten of 18 cases. Of the remaining seven cases, three showed moderate to strong staining on a proportion of tumour cells and four showed weak or absent staining on all cells.

**Figure 1** Case no. 18 stained with PA2.6 (monomorphic HLA-A,B,C). Negative tumour and positive stroma. Tu and st denote tumour and stroma respectively in this figure and subsequent figures except Figure 2a.

Although the expression of HLA-A,B,C free heavy chain (unassociated with β2-microglobulin) as detected by Mab HC-10 usually mirrored the expression of mature (β2-microblobulin associated) heavy chain as detected by Mab PA2.6, in cases 7, 10 and 13 free heavy chain was undetectable despite moderate to strong expression of mature heavy chain (Table I).

Of greater interest was the observation that there were patients whose tumour showed selective loss of some class I antigens. Selective loss was defined as complete absence of a specific HLA-A,B or C product from tumour cells in the presence of moderate to strong expression of monomorphic HLA antigens on tumour stroma. Because of uniformly weak or absent expression of all HLA-A and B antigens detected by monomorphic determinants, five of 18 cases could not be scored for selective loss. One of the remaining 13 tumours showed unequivocal selective loss confirmed by retesting on three separate occasions. This was one of the 12 tumours whose stroma was positive for Bw6. None of seven A-2 positive none of four A-3 positive and none of three Bw4 positive tumours showed selective loss (Table I, Figure 2a,b).

Partial degrees of selective HLA-A,B,C allele product loss are difficult to identify with certainty, though cases showing strong staining with monomorphic HLA-A,B,C Mabs but weak staining with some polymorphic Mabs (such as case 7) which demonstrate weak expression of Bw6 but strong expression of A-2 may be examples of this phenomenon. Using

**Figure 2** **a**, Case no. 13 stained with W6/32 (monomorphic HLA-A,B,C). Positive tumours and positive stroma. **b**, Case no. 13 stained with 126.39 (anti HLA-Bw6). Negative tumour and positive stroma.

**Figure 3** Case no. 11 staining with HB55 (anti HLA-DR). Negative tumour and positive stroma.

**Figure 4** Case no. 15 staining with TS 2/9 (anti LFA-3). Negative tumour and limited staining of stroma.

this lower definition of loss, only four of 18 could be said to have totally normal expression of HLA-A,B,C with the available Mabs against monomorphic and polymorphic determinants.

The pattern of antigen expression on tumour cells using Mabs specific for class II antigens was heterogeneous and often focal. Although nine of nine were positive for the 'core' HLA-D antigen recognised by Mab TU39. Seven of nine were positive for DR, five of nine positive for DQ and three of nine positive for DP. HLA-DP and DQ expression mirrored HLA-DR and in no case was a tumour observed to be HLA-DR negative yet HLA-DP or DQ positive. There was not any direct correlation between the expression of HLA-D antigens and that of degree of T cell infiltration (see below) into the tumour area. In one case (11) strong expression of HLA-DR antigen was observed on normal epithelium adjacent to HLA-DR negative neoplastic epithelium (Figure 3).

LFA-3 was expressed with moderate to strong intensity in 16 of 18 cases. There were two cases of complete LFA-3 loss (cases 15, 18, Table I, Figure 4) and in both cases the HLA-A,B,C antigens were either lost or down-regulated.

In a subset of the cases, the nature of tumour infiltrating lymphocytes was investigated using Mabs specific for different T cell subtypes. T cells (CD4>>CD8) positive were found to be present in tumour stroma (11 of 12 cases) the intra-epithelially (eight of 12).

### Discussion

Although post-mortem material is not ideal for such studies because of risk of autolysis and problems of induction of class II antigen by terminal bladder infection, the results do confirm the observations from larger studies (Gardiner *et al.*, 1985) that class I but not class II is expressed on normal usothelium.

Against this background the results of these tumour studies have demonstrated that HLA-A,B,C antigen expression is demonstrated in over a third of TCCs. Also, there was a definite case (13) of selective loss of HLA-Bw6 antigen in the presence of normal expression of other HLA-A,B,C products, two cases of complete loss of LFA-3 molecules (both of which also had either complete or partial loss of all class I antigens) and, finally, a single example where HLA-D antigen expression was observed on normal epithelium adjacent to negative neoplastic epithelium. All of these abnormalities in the expression of a immunoregulatory molecules could be capable of conferring a selective growth advantage to tumour cells by enabling escape from T cell immune attack directed against tumour specific antigens.

The selective loss of HLA-A,B,C allele products from neoplastic cells has previously been reported in other tumour types. These include Burkitt lymphoma (Masucci *et al.*, 1987) and colorectal adenocarcinoma (Smith *et al.*, 1989; Rees *et al.*, 1988; Momburg *et al.*, 1989). Such a selective loss of class I antigens could confer resistance to immune attacks against tumour, given the varying ability of different HLA-A,B,C allele products to present antigen epitopes to cytotoxic T cells. Thus, the loss of a single allele product could be functionally equivalent, in terms of escape from T cell attack, to the loss of all HLA-A,B,C molecules. Selective losses of HLA-A,B,C allele products could result from a single genetic mutation, whereas loss of all HLA-A,B,C antigens (e.g. case 18) would usually require more than a single mutation.

The incidence of colorectal adenocarcinomas with abnormal HLA-A,B,C antigen expression (11 of 30 cases) (Smith *et al.*, 1989) was similar to that reported in this study of bladder tumours. However, in contrast to the situation demonstratable in the bladder, most HLA-A,B,C loss in colorectal carcinomas was of individual HLA-A,B,C allele products and not a generalised loss of all HLA-A,B,C antigens as in bladder where there was an unexpectedly high frequency of loss of free heavy chain. This deserves further investigation.

The absence of detectable HLA-A,B,C free heavy chain in tumours expressing mature HLA-A,B,C antigen was seen in three cases (7, 10 and 13). The basis of this interesting observation may be a slow rate of HLA-A,B,C antigen synthesis by tumour cells.

Class II antigen expression as detected by TU39 Mab was often focal and of variable intensity on tumour cells of all nine case studies. Locus product specific Mabs for DR, DP and DQ also showed patch areas of positivity on the tumour cells. Similar results regarding the expression of class II antigens on neoplastic bladder epithelium were also reported by Gardiner et al. (1985).

The significance of class II antigen expression on bladder tumour cells is not clear, but it is conceivable that the detection of tumour antigen by infiltrating T cells results in the production of cytokines which in turn stimulate induction of tumour class II products. The presence of T cells in both tumour stroma and intra-epithelial areas is agreement with this hypothesis. Supportive evidence comes also from the observation that it has been possible to use interleukin-2 (IL-2) to expand activated T cells from six of 18 of these tumour biopsies using Il-2 after culture of tumour cell suspension (A. Nouri, in preparation).

The complete loss of LFA-3 molecules from tumour cells occurred in two of 18 cases. This may give tumour additional advantage to escape from immune surveillance given the importance of LFA-3 binding to CD2 molecules on CD8 cytotoxic cells to initate cytoxicity. The fact that LFA-3 loss is not, however, confined to bladder neoplasia, having been also demonstrated in colorectal adenocarcinoma (Smith et al., 1989) and Burkitt lymphoma (Gregory et al., 1988) would also support this observation. It is interesting to note that the two cases (15 and 18) showing LFA-3 losses also showed complete or major down-regulation of HLA-A,B,C antigens.

Given the focal nature of these antigen losses and the small size of tumour sample tested, it is obvious that the frequency of loss reported in this study is a minimum incidence.

Attempts were made to correlate the presence or absence of invasion (according to UICC system) with class I antigen expression on tumour cells. No significant association was demonstrated in the small numbers tested but it is of interest that the tumour not expressing any HLA-A,B,C antigen or LFA-3 (case 18), a further tumour not expressing LFA-3 (case 14) and the tumour showing the loss of HLA-Bw6 (case 13) were all invasive and no non-invasive tumour showed this degree of loss. More extensive testing is required to clarify whether loss of these immuno-regulatory molecules does lead to selective growth advantage to tumour.

This work was supported in part by Imperial Cancer Research Fund and The London Hospital Medical College Oncology Fund. We gratefully acknowledge the help of our clinical colleagues, Professor J.P. Blandy, Mr B. Jenkins and Dr J. Martin for help with provision and processing of material, Sir Walter Bodmer for helpful discussions and Elspeth Senior for help in preparation of the manuscript.

### References

ARCE-GOMEZ, B., JONES, E.A., BARNSTABLE, C.J. & others (1978). The genetic control of HLA-A and B antigens in somatic cell hybrids: requirement for β-microglobulin. *Tissue Antigens*, **11**, 96.

BERGER, A.E., DAVIS, J.E. & CRESSWELL, P. (1982). Monoclonal antibody to HLA-A3. *Hybridoma*, **1**, 87.

BRODSKY, F.M., PARHAM, P., BRANSTABLE, C.J., CRUMPTON, M.J. & BODMER, W.F. (1979). Monoclonal antibodies for analysis of the HLA system. *Immunol. Rev.*, **47**, 3.

COHEN, P.J., LOTZE, M.T., ROBERTS, J.R., ROSENBERG, S.A. & JAFFE, E.S. (1987). Immunopathology of sequential tumour biopsies in patients on IL-2. *Am. J. Pathol.*, **129**, 208.

FESTENSTEIN, H. & SCHMIDT, W. (1981). Variation in MHC antigenic profiles of tumour cells and its biological effects. *Immunol. Rev.*, **60**, 85.

FLEMING, K.A., MCMICHAEL, A., MORTON, J.A., WOODS, J. & MCGEE, J.O.D. (1981). Distribution of HLA class I antigens in normal human tissue and in mammary cancer. *J. Clin. Pathol.*, **34**, 779.

GARDINER, K.A., SEYMOUR, G.J., LAVIN., M.F., STUTTON, G.M., GEMMELL, E. & HAZAN, G. (1985). Immunohistochemical analysis of the human bladder. *Br. J. Urol.*, **58**, 19.

GOTCH, F., ROTHBARD, J., HOWLAND, K., TOWNSEND, A. & MCMICHAEL, A. (1987). Cytotoxic T lymphocytes recognise a fragment of influenza virus matrix protein association with HLA-A2. *Nature*, **326**, 881.

GREGORY, C.D., MURAY, R.J., EDWARDS, C.F. & RICKINSON, A.B. (1988). Down-regulation of cell adhesion molecules LFA-3 and ICAM-1 in Epstein-Barr virus-positive Burkitt's lymphoma underlines tumour cell escape from virus-specific T cell surveillance. *J. Exp. Med.*, **167**, 1811.

HUI, K., GROSVELD, F. & FESTENSTEIN, H. (1984). Rejection of transplantable AKR leukaemia cells following MHC DNA-mediated transformation. *Nature*, **311**, 750.

KRENSKY, A.M., ROBBINS, E., SPRINGER, T.A. & BURAKOFF, S.J. (1984). LFA-1, LFA-2 and LFA-3 antigens are involved in CTL-target conjugation. *J. Immunol.*, **132**, 2180.

LAMPSON, L. & LEVY, R. (1980). Two populations of Ia molecules on a human B cell line. *J. Immunol.*, **125**, 293.

MASUCCI, M.G., TORSTEINDOTTIR, S., COLOMBANI, J., BARUTBAR, C., KLEIN, E. & KLEIN, G. (1987). Down-regulation of class I HLA antigens and of the Epstein-Barr virus-encoded latent membrane protein in Burkitt lymphoma lines. *Proc. Natl Acad. Sci.*, **84**, 4567.

MCMICHAEL, A.J., GOTCH, F.M. & ROTHBARD, J. (1986). HLA-B37 determines an influenza A virus nucleoprotein epitope recognised by cytotoxic T lymphocytes. *J. Exp. Med.*, **164**, 1397.

MOMBURG, F., DEGENER, T., BACCHUS, E, MOLDENHAUER, G., HAMMERLING, G.J. & MOLLER, P. (1986). Loss of HLA-A,B,C and de novo expression of HLA-D in colorectal cancer. *Int. J. Cancer*, **37**, 179.

MOMBURG, F., ZIEGLER, A., HARPPRECHT, J., MOLLER, P., MOLDENHAUER, G. & HAMMERLING, G.J. (1989). Selective loss of HLA-A or HLA-B antigen expression in colon carcinoma. *J. Immunol.*, **142**, 352.

PAWELEC, G.P., SHAW, S., ZIEGLER, A., MULLER, C. & WERNET, P. (1982). Differential inhibition of HLA-D- or SB-directed secondary lymphoproliferative responses with monoclonal antibodies detecting human Ia-like determinants. *J. Immunol.*, **129**, 1070.

OLIVER, R.T.D. (1985). Biology of host/tumour cell interaction. In *Scientific Foundations of Urology*, Chisholm, G.D. & Williams, D.I. (eds) p. 624. Heinemann: London.

REES, R.C., BUCKLE, A.M., GELSTHORPE, K. & 4 others (1988). Loss of polymorphic A and B locus HLA antigens in colon carcinoma. *Br. J. Cancer*, **54**, 374.

ROSENBERG, S.A., LOTZE, M.T., MUUL, L.M. & others (1987). A progress report on the treatment of 157 patients with advanced cancer using lymphokine-activated killer cells and interleukin-2 or high dose interleukin-2 alone. *N. Engl. J. Med.*, **316**, 898.

SMITH, M.E.F., BODMER, W.F. & BODMER, J.G. (1988). Selective loss of HLA-A,B,C locus products in colorectal adenocarcinoma. *Lancet*, **i**, 823.

SMITH, M.E.F., MARSH, S.G.E., BODMER, J.G., GELSTHORPE, K. & BODMER, W.F. (1989). Loss of HLA-A,B,C allele products and lymphocyte function-associated antigen 3 in colorectal neoplasia. *Proc. Natl Acad. Sci. USA*, **86**, 5557.

SPITS, H., BORST, J., GIPHART, M., GOLIGANT, J., TERHORST, C. & DE VRIES, J.E. (1984). HLA-D can serve as restriction elements for human cytotoxic T lymphocytes. *J. Immunol.*, **14**, 299.

STAM, N.J., SPITS, H. & PLOEGH, H.L. (1986). Monoclonal antibodies raised against denatured HLA-B locus heavy chain permit biochemical characterization of certain HLA-C locus products. *J. Immunol.*, **137**, 2299.

WALLICH, R., BULBUC, N., HAMMERLING, G.J., KATZAV, S., SERGAL, S. & FELDMAN, M. (1985). Abrogation of metastatic properties of tumour cells be de novo expression of H-2K antigens following H-2 gene transfection. *Nature*, **315**, 301.

WATSON, A.J., DEMARS, R., TROWBRIDGE, I.S. & BACH, F.H. (1983). Detection of a noval human class II HLA antigen. *Nature*, **304**, 358.

ZINKERNAGAL, R.M. & DOHERTY, P.C. (1979). MHC cytotoxic T cells: studies on the biological role of polymorphic major transplantation antigens determining T cell restrictio specificity. *Adv. Immunol.*, **27**, 51.